# Developing Design Solutions for Smart Homes Through User-Centered Scenarios

**DOI:** 10.3389/fpsyg.2020.00335

**Published:** 2020-03-20

**Authors:** Mi Jeong Kim, Myung Eun Cho, Han Jong Jun

**Affiliations:** ^1^School of Architecture, Hanyang University, Seoul, South Korea; ^2^Construction Research Institute, Hanyang University, Seoul, South Korea

**Keywords:** smart home, user-centered scenario, design solution, framework for smart home service, context

## Abstract

The success of smart homes is fundamentally dependent on their adoption and use by people in the context of daily life. This study investigated ways to control and adapt the technology to fulfill user daily needs, which are the active drivers of smart housing technology adoption. A framework of smart home services was developed by focusing on the practicability of each variable from the perspective of supporting user experience. By developing scenarios based on previous studies, we identified residents’ behaviors and intentions regarding smart home technology and its use. Their issues were identified through the health problems and daily activities identified in the scenarios, and customized smart home services were developed for each resident based on technical solutions, space requirements, and design solutions. The main strength of this study is the adoption of user-centered methods to build a rich picture of the issues that affect households and the community related to the design, installation, and use of technology.

## Introduction

The development of information and communication technologies (ICTs), Internet of things (IoT), big data, and artificial intelligence (AI) has started changing people’s daily lives recently. Computing and information processing are spreading into daily life since these are increasingly being embedded in environments and artifacts invisibly. A new paradigm of human–computer interaction (HCI) is the integration of humans and humans, humans and objects, and objects and objects, and organically connecting them. This new technological paradigm is expected to cause significant changes in various fields, but it is predicted that the future information technology (IT) environment will be developed around the home (De Silva et al., 2012; Krishna and Verma, 2016; Borsekova et al., 2017). Noury et al. (2003) introduced the concept of a “health smart home” as a variation of smart homes with a focus on assistive technologies for the independence of the elderly and patients in housing (Noury et al., 2003). Many studies have developed techniques for specific groups of users, such as those with dementia, those vulnerable to falls, and those who would require emergency help. Numerous research projects have implemented a variety of prototypes of smart systems, which include sensors, algorithms, and intelligent devices (Das et al., 2002; Mihailidis et al., 2008; Krishna and Verma, 2016).

Existing smart home research has focused on technology development related to intelligent housing that can demonstrate new possibilities for the use of advanced technologies. These studies initially focused on home automation and networking technologies that facilitate remote control of electrical, lighting, and heating appliances (Arunvivek et al., 2015). Intelligence has recently become augmented and pervasive (Ricquebourg et al., 2006). Current research on smart homes has emphasized collection of contextual information about the domestic environment and its residents and provision of customized, automated supports (Singh et al., 2014). These studies focus on technology adoption and emphasize the need to provide user-friendly interfaces but regard the user as a passive agent and are essentially not focused on the user perspective. In other words, technology adoption was not based on clear user-centered understanding. For example, a home telehealth service, which incorporates ICT into the medical industry, will save medical costs for seniors who need chronic disease and health care and ensure independent living. Users perceived it as potentially useful, but in practice, they often refuse biosignal measurements and daily life monitoring through various sensing systems, such as cameras (Peek et al., 2014; Cimperman et al., 2016). This phenomenon is due to the introduction of technology without an in-depth understanding of its users.

Recently, the necessity of conducting smart home research in a more user-centered manner has been suggested, on realizing that technology development cannot achieve substantial results in other IT fields without a user-centered vision. The overall success of smart homes is fundamentally dependent on people’s adoption and use of this concept in the context of everyday life, regardless of the eventual form in which they adopt it. This study investigated ways to control and adapt the technology to fulfill users’ daily needs, which are the active drivers of smart housing technology adoption. To this end, we developed a framework of smart home services that focuses on the practicability of each variable from the perspective of supporting user experience. To validate this framework, this study captures each variable’s implications for smart home services through the proposed framework and proposed smart home services and solutions tailored to each individual based on the complex context of daily life. In particular, this study does not focus on smart technologies, but rather on the services in which they are installed and used. The analysis method used has been developed with an interest in determining the ways in which smart technologies can be used based on the user situation and needs.

## Related Works

### Development of Smart Homes

Berlo and Allen (1999) described a smart home as “a working environment which includes the technology to allow the devices and systems to be controlled automatically.” Emphasis is placed on intelligent dwellings with automatic control, including for lighting, climate, appliances, and security systems, such as access control and alarm systems. As home networking has developed with the availability of high-speed internet technology, such as asymmetrical digital subscriber line technology (ADSL), the smart home concept has been expanded by installing sensors in objects used daily and by enabling interworking with mobile devices. Recently, Balta-Ozkan et al. (2013) defined a smart home as “a residence equipped with a high-tech network, sensors and devices, and features that can be remotely monitored, controlled, and provide services that respond to the needs of its inhabitants.” The key to smart dwellings is the ability to automatically control dwelling facilities and devices from outside the dwellings. New technologies such as AI and the IoT can analyze the living patterns of residents and enable communication and information collection between smart devices, objects, and humans (Orwat et al., 2008; Arunvivek et al., 2015). Many of the new technologies that use various sensing systems, such as motion sensors and video cameras, are being developed to the extent that they can automatically support the user’s contextual awareness without the need to directly manipulate devices (Mann et al., 2001; De Silva et al., 2012).

Research on smart homes has been conducted in various fields, but thus far, most of these are in engineering and technical sciences domain (Wilson et al., 2015). For these studies, the goal of smart homes is to improve the quality of life of residents through automated devices, to enable them to live a safe, healthy, comfortable life independently (Gračanin et al., 2011). For example, MIT AgeLab has developed a technology-based home service that integrates into everyday life to improve well-being and safety. It developed this service after evaluating residents’ attitudes and needs related to various aspects, such as daily activities, social activities, mobility, safety, and nursing (Agelab, 2017). Further, a multidisciplinary team at the Georgia Institute of Technology, under its “Aware Home” project, constructed a three-story house to test and evaluate the engineering design of smart homes and identified users’ habits and behavior models through footprint detection technology. It also proposed techniques for fostering lasting bonds and social exchange between family members (Cory et al., 1998). Marikyan et al. (2019) emphasize energy consumption management and healthcare needs of aging users in terms of the services and context-led aspects that smart homes provide (Flynn et al., 2016). By enabling residents to monitor and control their energy supply against demand, they propose a novel and profound solution that reduces energy use and promotes environmental sustainability (Balta-Ozkan et al., 2014; Bhati et al., 2017).

Health smart home provides next-generation medical care for seniors by enabling their family and carers to remotely monitor the health of seniors through technology (Orr et al., 2006). Pervasive computing applications can be useful for predicting falls based on changes in gait. Intelligent devices in the home, from cell phones to furniture, picture frames, kitchen utensils, and toilets, are used to motivate residents to manage their diet, take medications, or continue exercising (Hudson and Cohen, 2003). Moreover, telemedicine technologies that connect patients with clinicians to monitor physiological signals, such as heart rate, through wearable devices or devices attachable to clothing or skin, or to manage chronic diseases at home, are becoming increasingly common (National Research Council et al., 2004). Thus, the future of computing for homes lies in creating a healthy, intelligent, interactive living environment (Do and Jones, 2012). Innovations in technology should be used to improve individual lives and develop human potential. A common concern of all age groups—not just the elderly or patients—is whether they would be able to live comfortably in their homes. Therefore, the research on smart homes needs to be extended by considering ways to improve the well-being of the middle-aged and younger age groups, thus moving beyond the present elderly- and patient-oriented research.

### Users and Acceptance of Smart Home Technology

Technology developers and researchers claim that advanced, applied knowledge will make our lives more comfortable. Their purpose is to support the daily lives of residents through technologies, such as those for energy management, security, monitoring, and detecting incidents (Yu-Ju et al., 2002; Gračanin et al., 2011). Despite this broad range of potential and assumed benefits of technology adoption, if we focus only on technological features, the technology can disappear before they are even incorporated into our lives (Cook, 2012). Thus, smart home research requires a sustained, systematic understanding of users because adopting smart technologies and incorporating these in everyday life are important for the success of smart homes (Haines et al., 2007). For the elderly with chronic or health disabilities, home telehealth services are expected to improve the quality of life in the home, reduce medical expenses, and provide independent living (Onor et al., 2008; Choi et al., 2018). These services include access to personal health information or records, remote patient monitoring, and chronic disease management. However, the elderly, the target population of smart technologies, do not understand new IT-based solutions and concepts and face special challenges in using these solutions (Cimperman et al., 2013).

In particular, home monitoring technologies are designed to support safe and independent living at home (Mihailidis et al., 2008). Monitoring technologies, such as systems for emergency response, fall detection, and health and physiological monitoring, provide a customized residential environment that tracks and records autonomously. However, research indicates that many users do not accept these technologies and have a high rate of device abandonment (Lund and Nygård, 2003). Their non-acceptance and non-usage may be regarded as the failure of smart home designs and operational procedures (Fisk, 1998). Therefore, for the successful realization of smart homes, it is critical to understand the factors that potential users consider important and necessary, and then decide on acceptable technologies and functions, rather than being concerned with technological performance in isolation. Courtney (2008) stated that our society sometimes neglects or ignores privacy as it stresses the need for technology. Demiris (2004) raised concerns regarding the use of technology in homes, such as privacy violations of older people, anxiety regarding the use of unfamiliar technology, and unnecessary surveillance. In particular, recent advances in home telehealth services include the transfer, management, and analysis of personal health data, which leads to concerns regarding security problems (Cimperman et al., 2016). Similar to other types of technology, smart home technology is only effective if the user accepts it and integrates it into daily life (Cimperman et al., 2013). Understanding users who are willing to adopt IT is important in IT design and implementation. Thus, this study attempts to answer the following questions: Who are the potential users of smart homes? What are the smart home technologies that these users need in their daily lives?

## A Framework for Constructing Smart Homes Services

**FIGURE 1:**
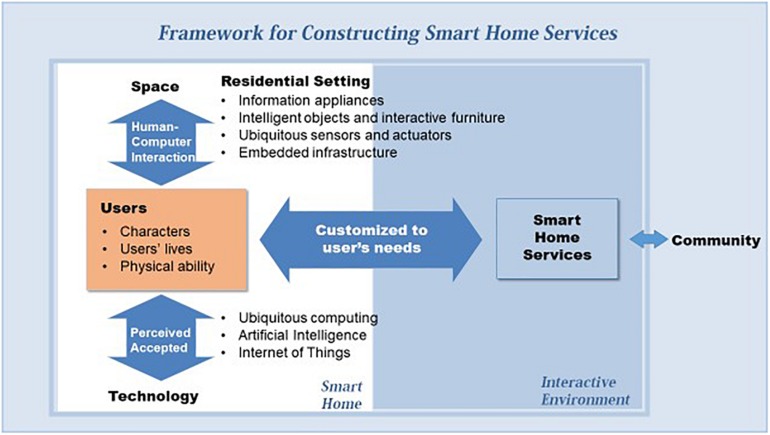
Framework for constructing smart home services.

This study proposed a framework to provide a structured way of understanding smart home services. The usefulness of frameworks is described in terms of three concerns: space, technology, and users. Figure 1 shows the framework for configuring smart home services. Unlike research that has focused on technical issues, the framework seeks to identify and integrate cross-cutting relationships based on understandings of smart homes and users. In particular, the framework’s focus is on multimodal interactions between users and smart homes that integrate space and technology. The space dimension focuses on HCI aspects, including user experience (UX), whereas the technology dimension emphasizes users’ perception and acceptance of technology. Intelligent computing and architecture are integrated to create new responsive and interactive environments. This environment is constantly connected to the network, where residents can interact with neighbors in the community to which they belong, and provides various residential services that are necessary and appropriate for residents. The proposed framework will help designers, architects, engineers, and researchers alike to explore and develop smart homes in a more expanded, integrated perspective. The framework of health smart home services, established on a framework constructed by Kim et al. (2014), is extended from a user and multidimensional perspective.

### Users

Smart homes must provide information and services tailored to the user’s situation. This is because residents in homes live differently, in accordance with their own characteristics and physical ability. Basically, people desire to gain increased comfort and convenience through the smart home, but the degree and method of realizing this goal can vary from individual to individual. This dimension has three categories related to user preference and experience: characteristics, lives, and physical ability. It is important to understand the characteristics and health of the user. For example, when a designer develops smart homes to assist individuals with dementia, the user’s needs need to be understood in detail not from the designer’s perspective, but from that of those individuals (Orpwood et al., 2005). To design a smart home that best meets users’ needs, we need to understand their lives in considerable depth. Crabtree and Rodden (2004) argued that smart home design might be informed by attending to the routines of the home; thus, the routines of the inhabitants’ everyday lives should be explicated. Groups of users will differ in terms of their wants, needs, and use of technology, and may require different design solutions. According to research results related to user characteristics, the younger the age, the higher the education level, and the higher the income, the more the use of technology, such as the internet and smartphone. Further, the greater the desire to continue living in the place where they live and the higher the intimacy with technologies, the greater the acceptance of smart housing technology (Peek et al., 2014).

The younger generation generally has more exposure to, and experience in using, technology than the older generation. Mihailidis et al. (2008) compared opinions and differences between generations to identify differences in technology preferences and acceptance among users. The higher the level of technical anxiety, the more hesitant the use of smart home technology; computer anxiety was found to inhibit the use of smart home technology by the elderly in particular (Holden and Karsh, 2010). The use of technology is related to issues such as security, privacy, and trust as well as practical and ergonomic concerns with user-friendliness. These issues present critical design challenges related to the interaction between the user and the smart home. People do not live alone, but rather, they share the same network connected to the community space and coexist and cohabit with others at the same time. Therefore, the concept of smart home service extends to the community and is required not only in personal life but also in community life. In this framework, users control and regulate technology more efficiently and become masters of their lives and smart homes.

### Space: Residential Setting

Domestic settings are different from work-oriented settings; further, smart home settings are unlikely to be purpose-built. Therefore, it is essential to understand housing in terms of activities, quality of life, and subjective happiness in the evolutionary context rather than in the contexts of work and efficiency. This dimension has four categories: appliances, household objects and furniture, sensor and actuators, and infrastructure. Rodden and Benford (2003) argued that home settings should be understood through diverse approaches that are concerned with functional forms of household artifacts and devices as well as interactive environments and technological infrastructures. Emphasizing the nature of space integrated with technology, Do proposed that a smart living environment is interactive and has sensors and actuators as part of the building, to support living in the forms of things that think, spaces that sense, and places that play (Do and Jones, 2012). To understand the context of home, different methods need to be adopted for collecting diverse aspects of the context and these aspects should be combined to create the complete context for the domestic dwelling. For example, ethnographic studies can highlight domestic routines, whereas longitudinal studies can identify the ways in which technologies are used (Crabtree et al., 2004). Ethnographic techniques can be developed to identify how people actually live with technology: the social, cultural and historical context; the participants’ experience of aging and ill-health; factors that matter to them; technologies in their home and life; and their capabilities to operate and interpret technologies.

Interactive technologies need to be manifested within the residential settings. Information appliances, such as internet fridges, perform a single function or cluster of functions with wireless networking (Gaver and Martin, 2000). Among interactive household objects, a reminder display creates a context for remembering, thus tempering the elderly’s memory loss, and a notice board can be combined with communication capabilities (Mynatt et al., 2000). The medication alarm system not only informs the time of taking the medication but also collects data on medication. Pressure and motion sensors tagged on slippers track weight, steps taken, or falls and send these data to medical staff (Hindus et al., 2001). Some furniture could act as interactive settings through sensors that detect actions, such as the DiamondTouch table and augmented cupboard (Crabtree and Rodden, 2004). A software platform can be applied to furniture or mirrors to provide a radio and weather and health information, or to automatically adjust the illumination according to the surrounding environment through sensors (Sponselee et al., 2008). Through such augmented appliances, objects, and furniture, residential settings can be transformed into interactive environments that effectively assist residents to live healthily at home. Reliable control methods are essential for efficiency in system operation, and thus, the design of HCI is a crucial component of intelligent settings in daily life. This includes designing computing diffused into homes to be unobtrusive, intuitive, and reliable to act in expected ways.

### Smart Technology

It is the age of ubiquitous and pervasive computing. The use of ICT is essential for smart dwellings because it changes daily lives in residences in meaningful, fundamental ways. ICT distributed in rooms, devices, and systems (i.e., lighting, heating, and ventilation) is aware of people’s activities and needs. This dimension has three categories: ubiquitous computing, AI, and IoT. IoT connects sensors, devices, actuators, radio frequency identification tags, laptops, and mobile phones to share network resources in conjunction with each other (Krishna and Verma, 2016). The technology helps in energy management systems and supports access to devices and remote monitoring of embedded devices (Chatzigiannakis et al., 2015; Li et al., 2016). Advanced AI not only collects occupants’ data but also applies visual and sensory-based tracking systems to identify them based on facial expressions and emotion recognition (Mano et al., 2016). Visual-based tracking systems, such as cameras, can monitor the status of occupants in the smart home. An AI-based IoT framework provides a continuous monitoring system of living patterns of residents through various sensors attached to the human body and in the environment to avoid health hazards and provide customized health care services accordingly (Mann et al., 2001).

Perceptual capability that is aware of the inhabitants and their needs is emphasized to provide customized and situated aids, and embedded intelligent components are used for context awareness. To establish intelligent infrastructures, various sensors need to be embedded in the fabric of the environments, which support drawing inferences from contextual information. Mihailidis et al. (2008) proposed home monitoring systems that are targeted toward specific home support goals: personal emergency response systems; automated fall detection; activity of daily living monitoring; environmental controls, such as lights, heating, and ventilation; and health monitoring, such as heart rate monitoring and detection of sudden changes in a person’s lifestyle patterns that may indicate changes in health, using sensors located in the environment (Mihailidis et al., 2008). Many studies have investigated monitoring of residents’ daily activities and physiological health conditions and described the state of the art of sensors, algorithms, and tracking devices in smart systems (Noury et al., 2003; Orwat et al., 2008; Marikyan et al., 2019). The smart technology framework monitors user mobility patterns and ensures a high level of functionality that preserves privacy and complementation of user data.

## Research Methodology

In this study, technologies and their use are considered in the context of the spaces of the home and community, and the networks of family and social relations linked to these technologies. We developed scenarios based on the results of the previous studies to build a rich picture of how people actually live in smart homes (Cho and Kim, 2017, 2018; Cho et al., 2018). Smart home design is an interrelationship between different disciplines. The importance of collaboration between specialty fields for solving the problems of existing smart home technologies and applying these solutions is becoming apparent. To find ways to effectively incorporate a comprehensive design for smart homes, 12 experts from various fields, such as IT developers, researchers, architectural designers, and employees from the IT services industry, the medical industry, and business enterprises, were invited to analyze and comment on scenarios. The scenario is fictional but based on the actual ethnographic account of the problems that people experience in residential dwellings. We encapsulated typical features and behavior of homogeneous subsegments of the target population. In designing the user experience, the most important trigger for effective decision-making is in-depth knowledge of users. The target groups of this study are the retired elderly in their 60s and 70s who live in their homes, those in their 40s and 50s who are actively socializing with their children, and those in their 30s who are growing rapidly. Three questions were provided for reflection: (1) What is important and what are the problems for the residents in each situation presented in the scenario? (2) How can space or technology improve their lives? (3) What are the smart home services that should be provided for residents in each situation?

### Scenarios

By developing scenarios, we identified residents’ behaviors and intentions as regards smart home technology and use. In particular, the scenarios focused on predicting the skills that people of different ages would need and use based on their health, work, and daily life experiences. A technology is always developed around standard users, and hence, the situation may not be suitable for other users. Eisma et al. (2004) suggested that developing technologies need to be built for diverse user groups and long-lasting relationships with them should be established. This article reports on an extensive study to engage with the cultural and contextual issues surrounding the use of new technologies by diverse users. The technical experiences of users and their physical ability and health status are important variables to consider in developing a smart home service. In this regard, previous studies (Cho and Kim, 2017, 2018; Cho et al., 2018) provide evidence that users’ needs and expectations differ according to age. Hence, as shown in Table 1, the scenarios we consider have three components: personal characteristics, physical ability and health condition, and daily home life and activities. Personal characteristics include age and gender. Physical ability and health conditions as well as daily home life and activities are based on the activities of daily living (ADLs), such as basic activities and instrumental activities, which help in understanding the physical health and daily activities of the user and in providing appropriate information and services. The first two scenarios in Table 1 consider a man and a woman, both aged 65 years. The next two involve a man, aged 52 years, and a woman, aged 45 years; both have children. The last two scenarios consider a man and woman aged 31 years and 38 years, respectively. These three types of scenarios are used to represent the age groups of 60–70, 40–50, and 30–40 years. However, further validation needs to be conducted for a broader population to generalize this study’s results.

**TABLE 1:** Six user scenarios.

**Resident type**	**Characters**	**Physical ability and health condition**	**Living and activities**
Elderly individual	Brian, 65 years old, male, retired, living alone	His blood pressure and thyroid levels increase, and hence, he continuously visits the hospital to check his levels and take medicine. His cognition and energy have weakened, and his appetite has worsened recently.	✓ He does not have many things to buy, and he always buys from the same shop. ✓ He finds house cleaning difficult. ✓ He is not used to touching smart devices and is afraid to learn something new. ✓ He goes to the senior welfare center by bus to be with his friends. ✓ He has meals in senior welfare centers and has only one meal at home.
	Jane, 65 years old, female, living with spouse	She had a shoulder surgery and has a lumbar disc. She regularly visits hospitals for treating her cataracts and low bone density.	✓ She rarely prepares meals at home and just cleans her house. ✓ She finds it very difficult to wash clothes. ✓ She misses her children, but finds it difficult to visit them, and sometimes they just talk on the phone. ✓ She goes to the hospital regularly but finds use of public transportation difficult because the hospital is far away. ✓ Shopping alone is difficult because she cannot see and hear well.
Middle-aged individual	Alex, 52 years old, male, office worker, living with spouse, a daughter, and a son	He is not very ill, but he has gastritis, high body fat, high cholesterol, and neck and waist disc problems because of his long sitting hours at work.	✓ As a manager of the company, he has frequent dinners and must work beyond office hours, and thus, he spends little time at home on weekdays. ✓ He sleeps on the weekends or watches television. ✓ His wife usually solves domestic problems, and he has little experience engaging in intimate conversations or activities with children. ✓ At home, he often checks his business or e-mail secretly on his cellphone and watches general news and banking news.
	Sarah, 45 years old, female, homemaker, living with spouse, and a daughter	She does not have a history of illness, but she tires easily and is weak because of lack of strength. Her eyes are aging, and she finds it difficult to read small print. When the weather is dry, her eyes or skin itch.	✓ She is not stylish and prefers practical items, such as comfortable shoes and clothes. ✓ She finds it difficult to push heavy carts or carry heavy loads when shopping. ✓ She knows she should exercise for health but finds it difficult to do so in practice. ✓ It is difficult for her family members to prepare food, clean ingredients, and clean and mop the house. ✓ She does not often stop by at her spouse’s family’s home or at her parents’ home and calls only occasionally.
Single individual	Paul, 31 years old, male, programmer, living alone	He is in good physical condition, but he has been concerned about his health.	✓ He often skips meals and eats irregularly. He sleeps in on weekends and has irregular sleeping times. ✓ He does not prepare food at home, and hence, he has no cooking ingredients. ✓ He often consumes fast food. ✓ He is not good at cleaning. ✓ Whenever he has time, he always watches videos on YouTube on his laptop. ✓ He is not active in sports and hobbies. ✓ He has no neighbors to know. ✓ He wants to succeed in the field he works in now.
	Suzan, 38 years old, female, office employee, living alone with a dog	She feels lonely. Her feelings are explosive and difficult to control, and she feels stressed.	✓ She usually buys a meal and is on a diet. ✓ When she cares, she often sleeps poorly or does not sleep well. ✓ She does not prepare food at home. She needs to clean, but she feels stressed when she sees a house that is not cleaned because she does not have time. ✓ She does not meet well with family or friends. ✓ She likes to talk on the phone with her friend or mother. ✓ She is busy with her work and comes home to sleep and not to play sports or hobbies.

### Proposed Smart Home Service According to Context-Based Solutions for Scenarios

The residents’ living problems were identified through their health problems, activities, and daily routines presented in the scenarios, and customized smart home services needed for each resident were developed based on technical solutions, spaces, and design solutions.

#### Identifying Problems Through Health Status and Daily Life Analysis

On analyzing the first scenario, that of elderly people in their 60s and 70s, experts pointed out the health problems caused by physical aging and the difficulties of performing household work, such as basic cleaning and washing, and basic purchasing activities such as for food items and daily necessities. They expressed concern about the elderly individuals’ lack of activity, the disconnection of their social relationships, and their difficulty in interacting with their children. The difficulty of attending hospital for regular medical care was also highlighted as a problem. It was also found that elderly people lack experience in using modern technology and their fear of devices could make technology adaptation difficult.

By analyzing the second scenario, which considered middle-aged people in their 40s and 50s, experts identified that in terms of health, middle-aged people were coping with stagnation, lack of strength, fatigue, and low motivation. It was perceived as a problem that this group lacked the time required to solve the problems of lack of rest and regular exercise and inability to have individual time and to engage in hobbies and community activities. The experts also identified that middle-aged individuals found it difficult to take good care of their health, since they led busy lives, and that they also found it difficult to communicate with their adolescent children.

In the third scenario, related to unmarried singles in their 30s, experts recognized mental health issues, such as loneliness and stress, as critical problems. In addition, irregular activities, such as skipping meals and inadequate sleep, were thought to be difficult factors in daily life. They pointed out that singles found cooking and cleaning bothersome and regard their home as a sleeping space. The problem is that their loneliness can increased because they do not know, or interact with, others in the neighborhood. Further, singles do not engage in enjoyable sports and hobbies. The experts identified issues and needs in each scenario, as shown in Table 2.

**TABLE 2:** Scenario analysis.

**Type of residents**	**First scenario: the elderly**	**Second scenario: the middle-aged**	**Third scenario: singles**
Issues	✓ Health problems ✓ Inconvenience of going to hospital for regular medical care ✓ Difficulties in household work ✓ Difficulty in shopping and buying necessities ✓ Low physical activity ✓ Social isolation ✓ Problems with children ✓ Fear of new technology and device adaptation	✓ Stamina decreases as aging begins ✓ No strength and tires easily ✓ Difficult to take care of health in their busy life ✓ Lack of rest because of continuous work at home ✓ Lack of regular activity ✓ Cannot afford personal time, hobbies, or community activities ✓ Lack of conversation with children	✓ Poor mental health, such as feeling lonely or stressed ✓ Irregularities in sleeping, eating, etc. ✓ Home care neglect, such as lack of cleaning and cleanliness ✓ No exchange between neighbors ✓ Lack of exercise and hobbies; uses home as a sleeping space
Needs	✓ Therapy and telemedicine ✓ Automation of daily routines ✓ Assist activities ✓ Family interaction ✓ Social implications ✓ Re-education about new technology	✓ Health care and consultancy ✓ Telework ✓ Rest ✓ Leisure and exercise ✓ Family connectivity	✓ Overcome the feeling of isolation ✓ Regular meals and sleep ✓ Cleaning and home management ✓ Social connection ✓ Community exercise and hobby

#### Solution of Technologies and Spaces for Smart Homes

To develop solutions, we considered the technical and spatial aspects of the problems. We summarized the technical parts into functions and devices and divided the spatial parts into unit households and communities within the complex (see Table 3 for details).

**TABLE 3:** Technical and spatial solutions.

**Type of residents**	**First scenario: the elderly**	**Second scenario: the middle-aged**	**Third scenario: singles**
**Technical solution**			
Function	Monitoring mobility Fall recognition Recognizing crisis Activity tracking and alarm Reminder Assistance Therapy delivery Telecommunication	Health care and management Health data repository Physiological monitoring Virtual exercise Remote business systems Public space reservation function (server, app support, etc.) Family schedule sharing	Remote access via mobile device Safety against theft and fire Control and monitor environment (heat, gas, electricity, and light) and appliance Assessment of abnormal sleeping patterns Social network of similar ages in complexes
Device	Helper robot Voice talker/Secretary Video call Medicine reminder	Intelligent appliance Virtual trainer: virtual reality exercise support program Health check and care smart device	Intelligent appliance Smart potted plants or smart pets Voice friends Sleep, eating, exercise alarm; virtual reality exercise equipment
**Spatial solution**			
Unit and design	Stretching zone Personal exercise space, medicine storage Color to give psychological stability Lighting that reduces eye strain Floor material for fall protection	Smart family room Smart home training room Automated kitchen facilities for easy food preparation and cooking Remote workspace Interactive furniture placement	Multipurpose space Sleep induction bed Flexible wall that can alter spaces
Common space	Seniors’ meeting space Gym for the elderly Promenade and vegetable garden Health measurement and treatment space	Athletic spaces, such as swimming pools and tennis courts Family break area Rest area, such as sauna and library Health measurement space	Restaurants for meals Community promotion space with night programs Party room for inviting friends

For the elderly in their 60s and 70s, the technical solution most mentioned was the need for continuous monitoring through sensors to identify everyday patterns and cope with crisis situations. In addition, technology acceptance was a problem, although they need technical help for managing chronic disease and regular treatment. The aspect of most interest for proposing spatial solutions was social exchange, and therefore, various community spaces within the complex were suggested, such as meeting spaces, exercise facilities, and paths for outdoor walks.

For those in their 40s and 50s, who are unable to take good care of themselves owing to a busy schedule, it is useful to continuously measure blood pressure and sugar levels and pulse rate as a technical solution, collect data, and provide health counseling, management, and exercise guidance. A smart home training room that has a spatially assisted exercise console was proposed. In addition, to build harmonious family relationships, a smart family room that combines the necessities of space and technical solutions was proposed. This is a space equipped with smart technologies, such as context awareness and augmented intelligence, and is a space specifically set aside for family-friendly programs, which expands the existing family room concept. For rest and self-time, a rest area, such as a sauna, a library, and a wine bar in the complex or a family rest area, is proposed.

In the case of singles in their 30s, when they leave their workplaces late, they often cannot clean or perform housework, and hence, through technology, it is necessary to monitor and control the house from other places. It was suggested that they can easily interact with each other through apps by forming social networks with others of similar ages in complexes and meet when needed for engaging in shopping, hobbies, and sports together. Spatial solutions are also required for a variety of activity spaces to share social activities, walks, hobbies, etc., and promote social life. For this generation, it is difficult to solve problems simply by providing space, and thus, it is necessary to develop various programs that can activate meetings and continue social exchanges, and various meeting apps that induce participation in the complex. This approach would resolve social disconnection and isolation of these individuals.

For people of all ages, the space for health measures, proper treatment, and customized exercise based on such treatment must be provided. Therefore, it is desirable to establish a professional nursing space in the complex to enable health measurement and data management for each individual and to provide simple rehabilitation treatments or exercise guidance in connection with community hospitals.

#### Customized Smart Home Services

Various smart home services can be presented based on problems and solutions identified in the three scenarios. In this study, smart home services are classified into five categories: basic daily life support, health care and management services, environment services, psychological well-being services, and social relationship enhancement services.

Basic daily life support is a service that helps residents with basic daily activities, such as household chores, shopping, and meal preparation. Specifically, this category includes services to perform household chores, such as laundry, washing dishes, and ironing; to enable shopping for, and delivery of, household essentials; and to prepare meals and side dishes to suit occupants’ needs. Devices, such as network information appliances and AI robots, can be used for automated data collection and storage of records of purchasing experiences. Based on user’s purchasing patterns, information and services (e.g., regular store automatic purchase) can be provided.

Health care and management services include hospital-based disease management, physiological measurement, health counseling, and exercise guidance services. Various sensors or devices in the house check and manage occupants’ health status of blood pressure and diabetes. Biomedical information collected along with health measurements through sensors or measuring instruments is provided in connection with community hospitals, and video-based medical consultations and medicine prescriptions are provided. It also provides health counseling based on residents’ health information and appropriate exercise guidance.

Environment services include security and safety, energy management, and cleaning services. It is provided to prevent, or cope with, safety accidents, such as theft and fire occurring in a house, or to reduce energy consumption, such as for lighting and heating. Cleaning services, such as for washing dishes, managing laundry, and cleaning the house, would be especially useful for seniors and singles.

Psychological well-being services are important for residents’ self-esteem, development, and happiness. Smart devices and internet education, technology installation and management, and smart entertainment services are included. The technology installation and management service is aimed at overcoming the frustration caused by inability to use technology and at improving usability along with technical education. It is related to establishing and installing the initial environment. It is a service that installs it for those who are not familiar with the new technology and helps in case of malfunction or failure. This can reduce the burden perceived on using unfamiliar technologies. Residents’ favorite entertainment such as videos, music, and games are managed, and customized information is provided in conjunction with AI.

Social relation enhancement services include those for communication, social connection, exercise, and hobbies in the community. Communication services are available not only in smartphones but also in intelligent objects, appliances, and walls with networks and platforms installed. Various channels and convenient interfaces promote interaction with family and friends at other locations. It provides information related to community facilities frequently used by residents in the area, supports various social activities, sports, and hobbies of residents through public spaces within the complex, and develops applications for program development, communication, and reservation of meeting space. The community program aims to eliminate the negative feelings of loneliness and to encourage residents to experience the happiness of being together by letting them participate and actively communicate in various fields, such as watching movies, reading, walking, and biking.

The services proposed by the smart home can be extended to various domains. Depending on residents’ particular situation, some services may be more important than others. Proposed smart home services are shown in Table 4. For the elderly, basic daily life support, health care and management, and social relation enhancement services are more useful than other services. Single individuals would find psychological well-being and social relation enhancement services more useful than others, whereas middle-aged people would need various services in all areas.

**TABLE 4:** Customized smart home services.

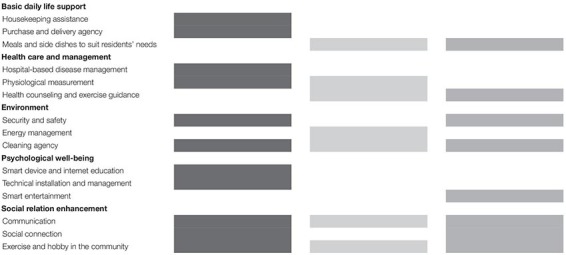

*The color shades show more important services for each age group.*

## Discussion and Conclusion

The purpose of this study is to develop and apply smart home services based on user-centered understanding for more practical and effective smart home implementation. The main strength of this study is the use of qualitative, user-centered methods to build a rich picture of the issues related to the design, installation, and use of technology that affect households and the community. This approach allowed us to develop detailed ideas and proposals to improve the daily lives of residents. Another strength of our study is its interdisciplinary nature—we incorporate the perspectives of those from various fields, including IT developers, researchers, architectural designers, and employees from the IT services industry, the medical industry, and business enterprises. Their diverse backgrounds allowed us to combine practical experience with theoretical approaches.

### Prospective Users and Challenges

One of the main goals of the early smart homes was to provide assistive services for people with disabilities and for the elderly. Recent trends in smart home research have highlighted healthcare services, and thus, activities and health-related assistance have become the most important goals of smart homes. However, the boundaries of smart home users and services need to be expanded. The dissemination of smart homes may be limited if they do not focus on actual needs. Smart technology is not exclusive, and innovations in technology should be used to improve individual lives and develop human potential, rather than being limited to the elderly or to patients. It is a common concern for people of all ages who want to live comfortably in their homes. The development of modular, affordable smart home technologies enables their incorporation into existing ones as well as newly built homes. The number of potential users will grow and may be women, men, and children of various generations living in homes. The challenge should be to gather data on a wider variety of residents and to be interested in their needs and their use of technology.

### Customization and Adaptation

The lives of residents in homes are not as repetitive and predictive as designers believe. People vary, and existing domestic environments are organic and dynamic. Technology may not be used in the way designers intended. As a result, it should be recognized that there is a need to consider the different situations of residents and try to solve the problem from various directions. It is desirable to focus on the benefits that technical assistance would provide and to identify the use and placement of appropriate technologies in the context of day-to-day life for a healthy and happy home. Tailored solutions for residents need to be provided in smart homes. The way in which the elderly, middle-aged, and singles live, and their state of health are inevitably different. Thus, the technologies, spaces, and services they need are very different. Different groups of users may require different design solutions in terms of aspects such as households, generations, and cultures. The important thing is to determine their needs, and then apply and adapt the demanded and preferred technologies for everyday life. There should be no social barriers to the adoption of smart homes, such as obstacles to providing effective, tailored services. Concerns regarding loss of control, reliability, privacy, trust, and irrelevance often make it difficult for individuals to accept smart home technology. We should understand the environment in which users can adopt technology and investigate whether and how smart home technologies may be effectively incorporated into the domestic context. The solution needs to be scalable, sustainable, and sociotechnical.

### Social Interaction and Support

Most services are applicable only to single smart homes, and sometimes to single rooms. Some research projects are devoted to location detection and do not implement any practical service for residents. We consider that if these limited systems with a few features are used, the smart home dream we imagine will not materialize. Without proper services and utilities, their widespread utilization cannot be achieved. The service area of smart homes should be expanded to satisfy residents. It is necessary to provide various services in connection with complexes and communities where residents live. Similar to the concept of telehospital/telemedicine service in conjunction with local hospitals, more new service networks might emerge that will connect homes for information sharing. The network serves as a platform for easy home access to services that are frequently used by residents, such as libraries, sports facilities, and welfare centers in communities. Future smart homes will promote the integration of all possible services into traditional homes. These homes will provide almost all the essential services, such as communication, medical, energy, public facilities, entertainment, and security services. In this study, we proposed a service that can be used by extending the concept to the community, but additional research is required to make these services cost-effective, efficient, and acceptable.

### Knowledge Sharing and Collaboration

The research on smart homes constitutes an interdisciplinary domain. The architecture of a smart home depends on other branches, such as technologies, spaces, and services. Smart homes benefit from improvement and diffusion through the integration of these sectors. While research on smart homes is typically conducted in the engineering, technology science, and design domains, there is increasing interest in various sectors ranging from healthcare, services, and economics to energy. In this study, we identified the need for knowledge sharing and collaboration in related fields. Many other methods are being developed in individual projects or research environments, and hence, smart home residential service chains lack effective integration and information sharing. It is time for an integrated approach to smart homes that focuses on users.

## Data Availability Statement

Publicly available datasets were analyzed in this study. This data can be found here: http://www.khousing.or.kr/.

## Author Contributions

MK and MC composed this study, designed the framework and the methodology part of the research, and completed the qualitative analysis. MK and HJ provided supervision throughout the research.

## Conflict of Interest

The authors declare that the research was conducted in the absence of any commercial or financial relationships that could be construed as a potential conflict of interest.
